# ERK5 MAP Kinase Regulates Neurogenin1 during Cortical Neurogenesis

**DOI:** 10.1371/journal.pone.0005204

**Published:** 2009-04-13

**Authors:** Paige Cundiff, Lidong Liu, Yupeng Wang, Junhui Zou, Yung-Wei Pan, Glen Abel, Xin Duan, Guo-li Ming, Chris Englund, Robert Hevner, Zhengui Xia

**Affiliations:** 1 Department of Pharmacology, University of Washington, Seattle, Washington, United States of America; 2 Department of Environmental and Occupational Health Sciences, University of Washington, Seattle, Washington, United States of America; 3 Graduate Program in Molecular and Cellular Biology, University of Washington, Seattle, Washington, United States of America; 4 Graduate Program in Neurobiology and Behavior, University of Washington, Seattle, Washington, United States of America; 5 Institute for Stem Cell and Regenerative Medicine, University of Washington, Seattle, Washington, United States of America; 6 Institute for Cell Engineering, Departments of Neurology and Neuroscience, Johns Hopkins University School of Medicine, Baltimore, Maryland, United States of America; Katholieke Universiteit Leuven, Belgium

## Abstract

The commitment of multi-potent cortical progenitors to a neuronal fate depends on the transient induction of the basic-helix-loop-helix (bHLH) family of transcription factors including Neurogenin 1 (Neurog1). Previous studies have focused on mechanisms that control the expression of these proteins while little is known about whether their pro-neural activities can be regulated by kinase signaling pathways. Using primary cultures and *ex vivo* slice cultures, here we report that both the transcriptional and pro-neural activities of Neurog1 are regulated by extracellular signal-regulated kinase (ERK) 5 signaling in cortical progenitors. Activation of ERK5 potentiated, while blocking ERK5 inhibited Neurog1-induced neurogenesis. Furthermore, endogenous ERK5 activity was required for Neurog1-initiated transcription. Interestingly, ERK5 activation was sufficient to induce Neurog1 phosphorylation and ERK5 directly phosphorylated Neurog1 *in vitro*. We identified S179/S208 as putative ERK5 phosphorylation sites in Neurog1. Mutations of S179/S208 to alanines inhibited the transcriptional and pro-neural activities of Neurog1. Our data identify ERK5 phosphorylation of Neurog1 as a novel mechanism regulating neuronal fate commitment of cortical progenitors.

## Introduction

During mammalian cortical neurogenesis, neuronal cell fate specification is dependent on the temporal and spatial expression of the bHLH family of transcription factors including Neurog1, Neurogenin 2 (Neurog2), and Ascl1 (Mash1) [1]–[10]. These transcription factors specify neuronal phenotype at the expense of glial fate and subsequent choice of sub-neuronal phenotypes during cortical development (glutamatergic vs. GABAergic). For example, although there may be a high degree of redundancy between Neurog1 and Neurog2 [11], [12], both are expressed in the dorsal telencephalon and direct multi-potent cortical progenitors to a pyramidal, glutamatergic neuron fate. Ascl1 directs cortical progenitors to a GABAergic neuron fate [9], and its expression is high in the ventral telencephalon but low in the dorsal telencephalon [12]–[14]. The pro-neural Neurog1 and Neurog2 induce the expression of NeuroD1, NeuroD2, and Nex, members of the NeuroD family of bHLH transcription factors, which induce terminal differentiation of the committed precursors into mature neurons.

It has been postulated that in addition to the intrinsic molecular properties of these bHLH transcription factors, extracellular factors present in the microenvironment may also influence the cell fate choice of progenitors [15]–[17]. Thus, it is conceivable that, in addition to the intrinsic induction of expression of Neurog1/Neurog2/Ascl1 proteins, extrinsic factors could activate protein kinase signaling pathways and modulate the pro-neural activity of Neurog1/Neurog2/Ascl1 via protein phosphorylation. However, most research so far has focused on understanding the transcriptional regulation of these bHLH transcription factors; there is currently limited evidence that their transcriptional activities or their ability to specify neuronal commitment are regulated post-translationally.

We recently reported that the ERK5 (Mapk7), a member of the mitogen-activated protein (MAP) kinase family, provides an instructive signal to specify cortical progenitors to a neuronal fate [18]. In this study, we tested the hypothesis that the pro-neural activity of Neurog1 may be regulated by ERK5 during cortical neurogenesis.

## Results

### Activation of ERK5 potentiates while inhibition of ERK5 attenuates Neurog1-stimulated neurogenesis

**Figure 1:**
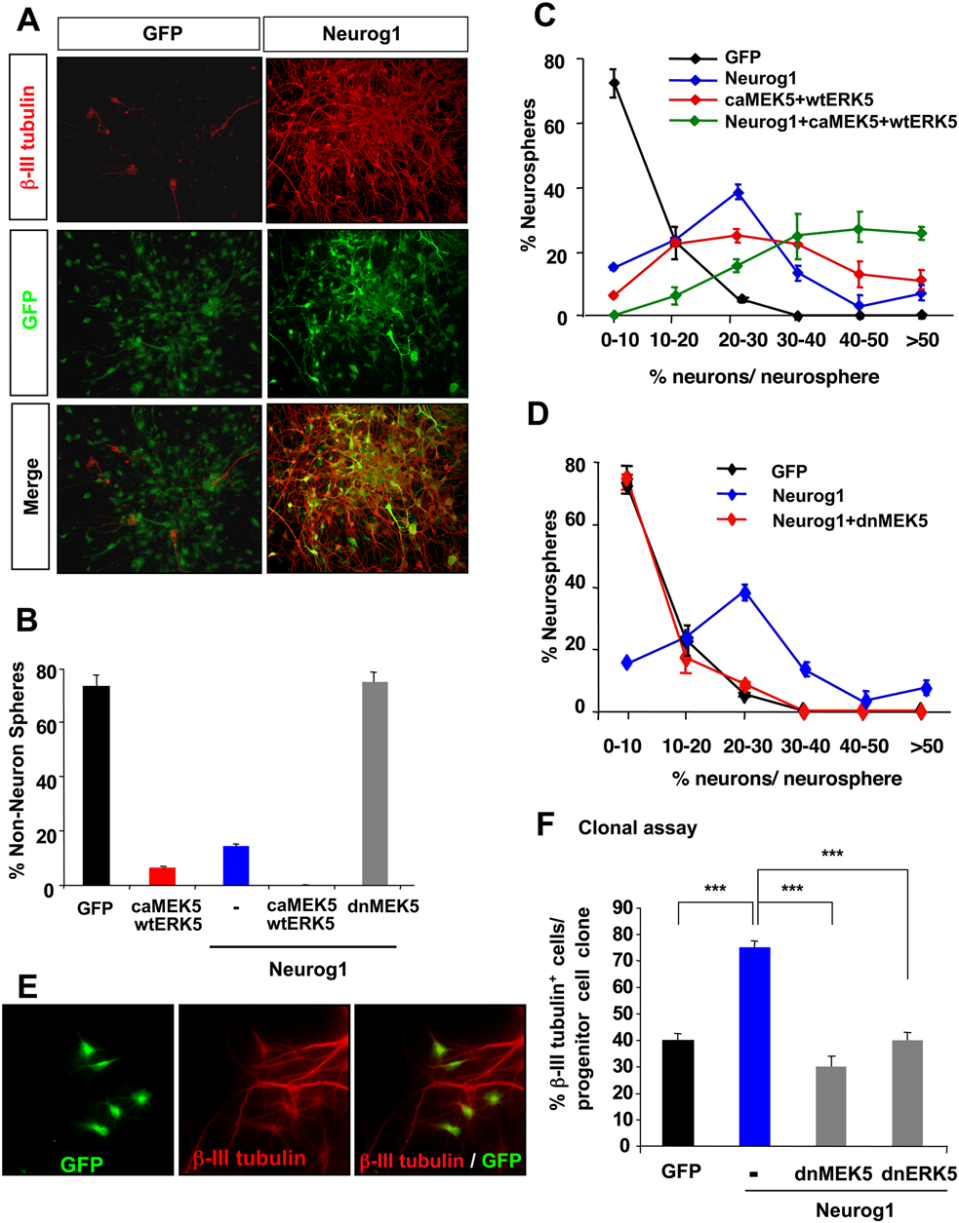
Activation of ERK5 potentiates while inhibition of ERK5 attenuates Neurog1-stimulated neurogenesis. For panels *A–D*, neurosphere assays. Freshly dissociated E13 cortical progenitors were co-infected with lentiviruses encoding Neurog1, constitutive active (ca) or dominant negative (dn) MEK5, or wild-type ERK5 as indicated. Cells infected with GFP-virus were used as a control. Neurospheres were allowed to form in culture for 5 d, and then transferred to PDL/laminin coated plates in bFGF-free medium to promote spontaneous differentiation for 3 d. Neurospheres infected with lentiviruses were identified by GFP expression. Neurons were identified by the pan-neuronal marker, β-III tubulin. *A,* Representative images of neurospheres infected with either GFP control virus (control) or wild-type Neurog1, and immunostained for β-III tubulin (red) and GFP (green). *B,* Effect of Neurog1 and ERK5 on the percentage of non-neuron spheres, defined as those neurospheres containing ≤10% neurons per sphere. *C,* Activation of ERK5 signaling potentiates the neurogenic effect of Neurog1. Data show distribution of the percentage of neurons per neurosphere. Data were collected from three independent experiments (n = 3). *D*, Inhibition of ERK5 signaling by dnMEK5 abolishes the neurogenic effect of Neurog1. *E,* Representative images of a progenitor cell clone in an adherent culture clonal assay, which allows us to specifically follow the cell fate of a single LeX^+^ cortical progenitor cell (Liu et al., 2006). Progenitor cells infected with lentiviruses were identified by GFP expression. Cells were immunostained for GFP (green) and β-III tubulin (red). *F,* Expression of dnMEK5 or dnERK5 suppresses the pro-neural effect of Neurog1 using the adherent culture clonal assay.

Our previous studies established that ERK5 is necessary and sufficient to promote neuron fate specification of cortical progenitors [18]. Because ERK5 is a MAP kinase that can phosphorylate and regulate the activity of several transcription factors, and Neurog1 can direct cortical progenitors to commit to a neuronal fate, we postulated that the pro-neural activity of ERK5 may be due to ERK5 regulation of Neurog1. To test this hypothesis, we performed a neurosphere assay to determine if ERK5 regulates Neurog1-stimulated neurogenesis *in vitro* **(** **Fig. 1** **).** Freshly dissociated embryonic day (E) 13 rat cortical progenitor cells were infected with lentiviral stocks encoding Neurog1, wild-type (wt) ERK5, constitutive active (ca) or dominant negative (dn) MEK5, an upstream kinase of ERK5. These genes were coupled to GFP through an internal ribosomal entry site (IRES) so that virus-infected cells can be easily identified by GFP expression [18]. Lentiviral infection was carried out 3 h after initial plating when the cells were still at the single-cell level in suspension. Neurons were identified by immunostaining of β-III tubulin, a marker expressed in immature neurons (**Fig. 1 A** **)**. Those neurospheres with less than 10 β-III tubulin^+^ cells were defined as non-neuron spheres. Quantification of the data demonstrated that ectopic expression of Neurog1 significantly reduced the total number of non-neuron spheres compared to control GFP-infected spheres (Neurog1 15%; GFP 73%) **(** **Fig. 1 B** **)**. This is consistent with other reports that ectopic expression of Neurog1 is sufficient to induce neurogenesis [6], [7]. Similar results were obtained with ectopic ERK5 activation (caMEK5+wtERK5), consistent with our previous report [18]. Co-expression of Neurog1 with caMEK5+wtERK5 generated no non-neuron spheres. Furthermore, the neurogenic effect of Neurog1 was reversed by co-expression of dnMEK5 which blocks ERK5 activation.

We next performed a more detailed analysis to examine the effect of ERK5 on Neurog1-induced neurogenesis by scoring the percentage of neurons within each neurosphere. In control GFP virus-infected neurospheres, the majority of the neurospheres contained ≤10% β-III tubulin^+^ neurons in each sphere **(** **Fig. 1 C** **)**. Expression of lentiviral Neurog1 greatly increased the number of neurospheres with a higher percentage of neurons. Activation of ERK5 signaling (caMEK5+wtERK5) also increased the number of neurospheres with a higher percentage of neurons. Significantly, ERK5 activation potentiated Neurog1-induced neurogenesis. In contrast, Neurog1-induced neurogenesis was completely blocked by inhibiting ERK5 signaling with dnMEK5 **(** **Fig. 1** D **)**. In addition to the neurosphere-forming assay, we utilized a progenitor cell clonal assay under adherent culture conditions [18] to examine the effect of ERK5 on Neurog1 at the single progenitor cell level **(** **Fig. 1 E** **).** Expression of dnMEK5 or dnERK5 blocked cortical neurogenesis stimulated by Neurog1 in this clonal assay **(** **Fig. 1 F** **).** These data provide evidence that the ERK5 signaling pathway regulates the pro-neural activity of Neurog1.

### ERK5 signaling regulates Neurog1-initated gene expression

**Figure 2:**
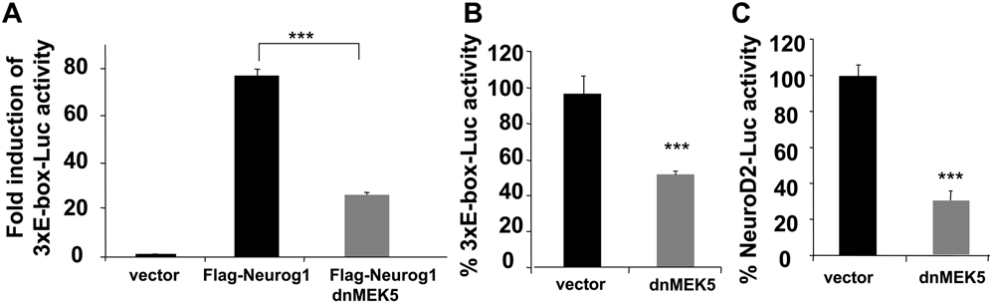
ERK5 signaling is required for transcription initiated by ectopically expressed Neurog1 and endogenous bHLH transcription factors in E13 cortical progenitors. Rat E13 cortical progenitor cells were transiently transfected with a control vector (vector), Neurog1, and dnMEK5 as indicated. The transcriptional activity of Neurog1 was monitored using a co-transfected 3xE-Box-Luc or a NeuroD2-Luc reporter. Luciferase activity was normalized to a co-transfected LacZ reporter. *A,* Expression of wt Neurog1 stimulates 3xE-box luciferase activity, which was inhibited by dnMEK5. *B*, Expression of dnMEK5 inhibits 3xE-box luciferase activity afforded by endogenous bHLH transcription factors. *C*, NeuroD2-luciferase activity induced by endogenous bHLH transcription factors is inhibited by dnMEK5.

Since Neurog1 induces neuronal cell fate specification and differentiation primarily through the transactivation of neuron-specific genes, we investigated if ERK5 regulates the transcriptional activity of Neurog1. Using a Nucleofector® method, we transiently transfected freshly isolated, rat E13 cortical progenitor cells with a Flag-Neurog1 expression vector and dnMEK5 as indicated **(** **Fig. 2** **).** Cells were co-transfected with a luciferase reporter construct driven by a Neurog1-reponsive, 3-tandem repeats of the putative E-box DNA binding site (CAGATG) (3xE-box-Luc). Ectopic expression of Neurog1 increased transcription initiated from the 3xE-box-Luc; this induction was significantly inhibited by co-expression of dnMEK5 (**Fig. 2 A**)**.** These data suggest that interfering with ERK5 signaling can disrupt gene transcription initiated by Neurog1.

Does ERK5 signaling modulate the activities of endogenous bHLH transcription factors? Although Neurog1, Neurog2, and Ascl1 are capable of binding and activating the E-box and native NeuroD2 promoter [6], [19], Ascl1 expression is extremely low in dorsal telencephalon [12]. Therefore, Neurog1 and Neurog2 are most likely the main endogenous transcription factors capable of stimulating the E-box-Luc or NeuroD2 promoter-driven reporters in cortical progenitor preparations isolated from E13 rat dorsal telencephalon. We transiently transfected E13 cortical progenitors with the 3xE-box-Luc or a NeuroD2 promoter-driven luciferase (NeuroD2-Luc) without introducing exogenous Neurog1 to monitor the activity of endogenous bHLH transcription factors. Cells were co-transfected with dnMEK5 to block ERK5 signaling or the cloning vector as a control. Inhibition of ERK5 signaling significantly reduced transcription of both reporters initiated by endogenous bHLH transcription factors present in rat E13 cortical cells **(** **Fig. 2, B and C** **).** Together, data in Figure 2 suggest that ERK5 regulates the transcriptional activity of Neurog1.

### ERK5 regulation of Neurog1 transcriptional activity may be mediated through phosphorylation

**Figure 3:**
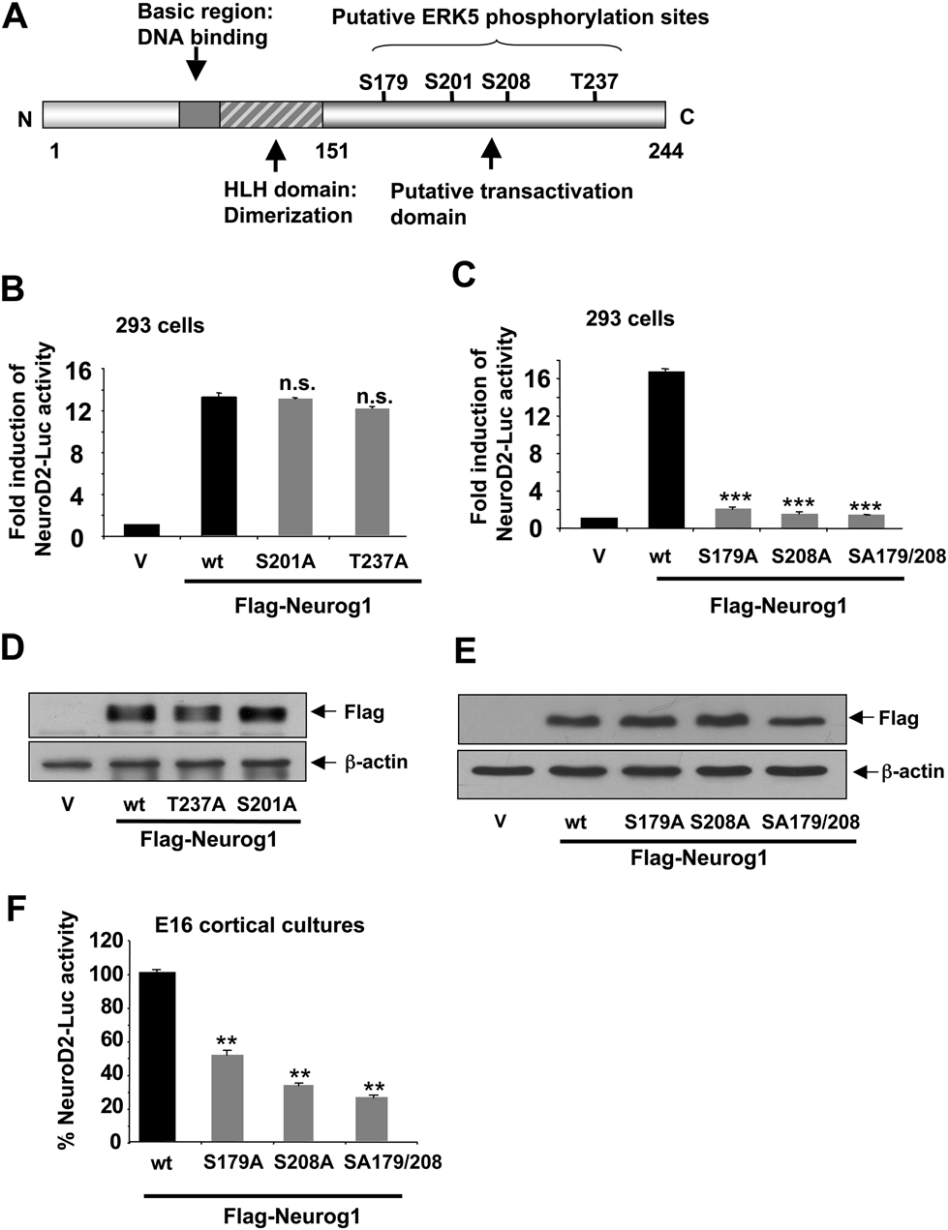
S179 and S208 are required for Neurog1's transcriptional activity. *A,* Schematic representation of the various functional domains of Neurog1. Four putative proline-directed MAP kinase phosphorylation sites (PX_1-2_S/TP), S179, S201, S208 and T237 are present within the presumed transactivation domain in the C-terminus. *B*, Replacing S201 or T237 with alanine had no effect on Neurog1's ability to stimulate NeuroD2-luciferase in HEK293 cells. V: vector control. *C*, Replacing S179, S208, or both with alanine almost completely abolished Neurog1's ability to stimulate NeuroD2-luciferase in HEK293 cells. *D, E,* Western analysis demonstrating equal expression of wt Neurog1 and Neurog1 mutants in HEK293 cells. *F*, Replacing S179, S208, or both with alanine greatly attenuates Neurog1's ability to stimulate NeuroD2-luciferase activity in E16 cortical neuron cultures.

Because ERK5 is a MAP kinase that can directly phosphorylate and regulate the activity of transcription factors [20], we postulated that ERK5 may regulate the transcriptional activity of Neurog1 through direct phosphorylation. A protein sequence analysis revealed two perfectly matched, putative proline-directed MAP kinase phosphorylation sites (PX _1-2_ S/T P), S179 and S208, and two imperfect sites (S/T P), S201 and T237, within the C-terminus of Neurog1 **(** **Fig. 3 A** **)**. Although mutations of S201 or T237 to non-phosphorylatable alanines had no effect on Neurog1's transcriptional activity **(** **Fig. 3 B** **),** replacing S179 or S208 with alanines almost completely abolished Neurog1's ability to initiate transcription in HEK293 cells **(** **Fig. 3 C** **)** and in cortical neurons **(** **Fig. 3 F** **)**. The distinct effects of the four mutations on Neurog1's transcriptional activity were not due to differential expression of the mutant proteins **(** **Fig. 3, D and E** **).** These results suggest that Neurog1's transcriptional activity requires the function of S179 and S208. Furthermore, phosphorylation of S179 and S208 may regulate the transcriptional activity of Neurog1. Because the double mutant SA179/208 was as effective as, if not more potent than, the single mutants we focused our efforts on the double mutant for the remaining investigation.

**Figure 4:**
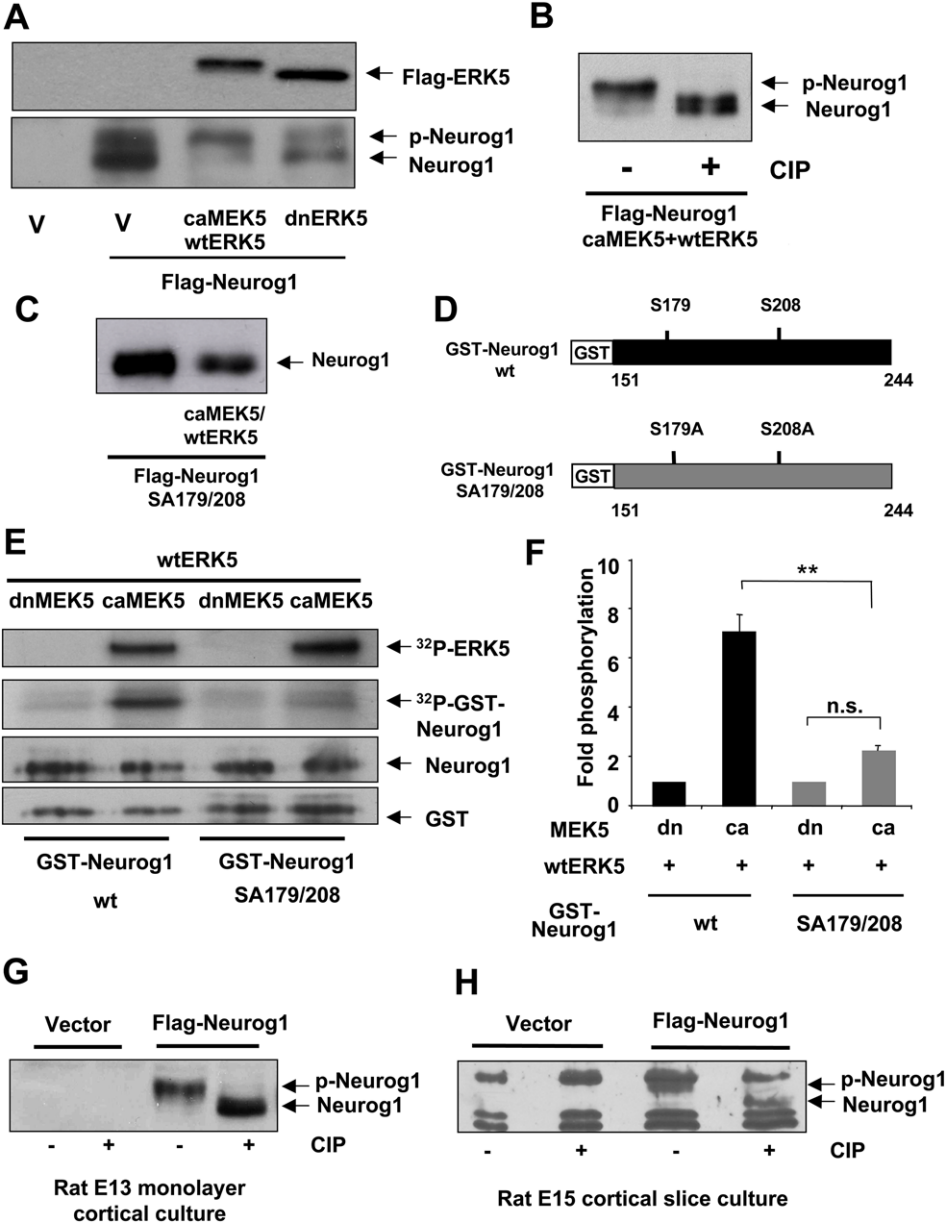
Activation of ERK5 is sufficient to induce Neurog1 phosphorylation. *A,* ERK5 activation in HEK293 cells leads to an electrophoretic mobility shift of Neurog1, indicative of Neurog1 phosphorylation (p-Neurog1). HEK293 cells were transiently transfected with either vector control (V) or Flag-Neurog1. Cells were also co-transfected with HA-tagged caMEK5 and Flag-wtERK5 to activate ERK5 signaling in transfected cells. Cells co-transfected with Flag-dnERK5 were used as a control. Cell lysates were analyzed by anti-Flag Western blotting. *B,* The electrophoretic mobility shift of Neurog1 was abolished after treatment with CIP. *C,* ERK5 activation does not induce an electrophoretic mobility shift of the mutant SA179/208 Neurog1. *D*, Schematic drawings of GST-Neurog1 fusion proteins in which the putative transactivation domains of the wt or SA179/208 mutant Neurog1 (a.a. 151–244) were fused with GST. *E*, Activated ERK5 can directly phosphorylate recombinant wt GST-Neurog1 but not the mutant GST-Neurog1 SA179/208 *in vitro*. Active ERK5 was immunoprecipitated using an anti-Flag antibody from HEK293 cells co-transfected with HA-tagged caMEK5 and Flag-tagged wtERK5. Cells co-transfected with dnMEK5 and wtERK5 were used as a control. The ability of ERK5 to directly phosphorylate Neurog1 (^32^P-GST-Neurog1) was measured by an *in vitro* kinase assay using recombinant GST-Neurog1 fusion proteins and ^32^P-ATP as substrates. ERK5 autophosphorylation (^32^P-ERK5) confirms the relative activity of ERK5. Western blotting for Neurog1 or GST was used to confirm comparable loading of the recombinant Neurog1 protein in the kinase assay. *F,* Quantification of data in panel E. Relative Neurog1 phosphorylation was normalized to Neurog1 protein levels based on anti-Neurog1 Western blotting. *G,* Ectopically expressed Neurog1 is phosphorylated in cultured rat E13 cortical progenitors. E13 cortical progenitor cultures were infected with lentiviral Neurog1 and maintained in bFGF-containing medium for 3 d. *H,* Ectopically expressed Neurog1 is phosphorylated in rat E15 *ex vivo* cortical slice cultures. Plasmid DNA encoding control vector or wt Flag-Neurog1 was electroporated *ex vivo* into the dorsolateral telencephalon of rat E15 brain. A GFP expression vector was co-electroporated to identify transfected region. Cortical slices were sectioned coronally and cultured for 40–50 h. GFP^+^ regions were excised out under a fluorescent microscope for Western analysis. To increase the yield of Neurog1 protein expression, a cocktail of proteasomal inhibitors and pan-caspase inhibitors were added to cultured cells or slices 6 h before harvesting in panels A–C, G, and H.

We investigated if activation of ERK5 induces Neurog1 phosphorylation. When Flag-Neurog1 was expressed alone in HEK293 cells or when co-transfected with dnERK5 as a control, it appeared as multiple bands on a 12% SDS gel, running at approximately 37 kDa **(** **Fig. 4 A** **)**. However, when co-transfected with caMEK5+wtERK5 to activate ERK5 signaling in transfected cells, the majority of the Flag-Neurog1 exhibited reduced electrophoretic mobility suggesting that Neurog1 is phosphorylated in cells when ERK5 is activated. Indeed, the reduced electrophoretic mobility of Neurog1 was abolished when whole cell lysates were treated with calf intestine alkaline phosphatase (CIP) **(** **Fig. 4 B** **)**, confirming that the gel shift is due to phosphorylation of Neurog1. In contrast, ERK5 activation did not reduce the electrophoretic mobility of the mutant SA179/208 Neurog1 (**Fig. 4 C** **).** Thus, activation of ERK5 signaling leads to wt, but not the mutant SA179/208 Neurog1 phosphorylation in HEK293 cells.

To determine if ERK5 directly phosphorylates Neurog1, active ERK5 was immunoprecipitated using an anti-Flag antibody from HEK293 cells transfected with HA-tagged caMEK5 and Flag-tagged wtERK5. The immunoprecipitated ERK5 was incubated with ^32^P-ATP and purified recombinant GST-Neurog1 (151–244) fusion protein **(** **Fig. 4 D** **)** as substrates in an *in vitro* kinase assay. HEK293 cells were also co-transfected with HA-tagged dnMEK5 and Flag-tagged wtERK5 as a control for the active ERK5. The kinase activity of ERK5 was monitored by its autophosphorylation (^32^P-ERK5) **(** **Fig. 4 E** **).** The wild-type GST-Neurog1 (151–244) was robustly phosphorylated by active ERK5 but not by the control inactive ERK5 **(** **Fig. 4, E and F** **).** Importantly, active ERK5 did not significantly phosphorylate the GST-Neurog1 SA179/208 mutant protein **(** **Fig. 4, E and F** **)**. These data suggest that ERK5 directly phosphorylates Neurog1 on S179, S208, or both.

To investigate if Neurog1 phosphorylation occurs in rat E13 cortical progenitors, freshly dissociated E13 rat cortical cells were infected with lentiviral stocks encoding GFP control or wt Neurog1. Cell lysates were collected 3 d later and treated with CIP **(** **Fig. 4 G** **)**. Treatment with CIP reduced the electrophoretic mobility of Neurog1, indicating that Neurog1 expressed in E13 cortical progenitor cells exists as a phosphorylated protein. Similarly, Neurog1 was phosphorylated when expressed *ex vivo* in rat E15 cortex slices **(** **Fig. 4 H** **).**

### Phosphorylation of Neurog1 at S179 and S208 regulates the pro-neural activity of Neurog1

**Figure 5:**
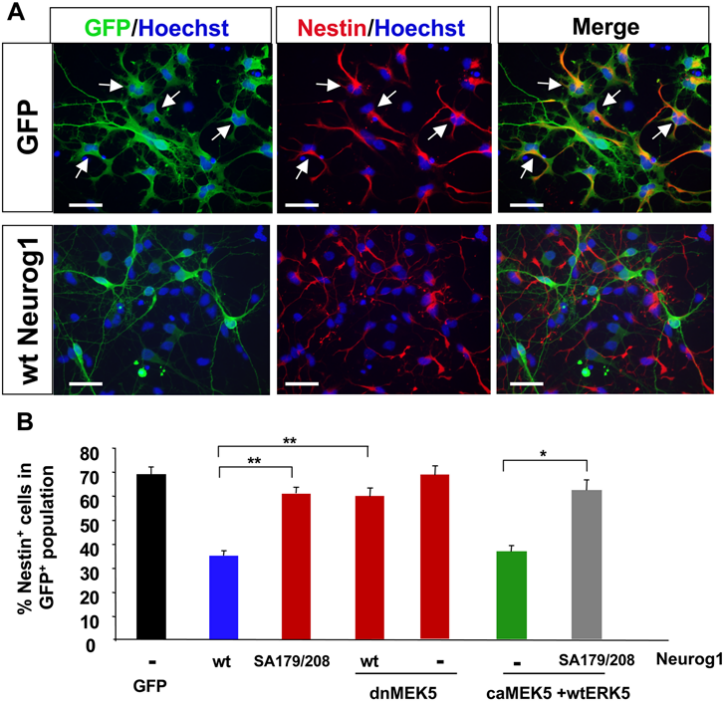
Wild type, but not SA179/208 mutant Neurog1 reduces the pool of nestin^+^ cortical progenitors in monolayer cortical progenitor cultures. Freshly dissociated, LeX^+^-sorted E13 rat cortical progenitors were plated on PDL/laminin-coated plates and immediately infected with various lentiviral stocks encoding genes of interest-IRES-GFP as indicated. Cells were grown for 2 d in bFGF containing medium, and then in bFGF-free medium for another 2 d to allow differentiation. *A,* Representative deconvolution images of cells infected with either GFP control virus or wt Neurog1, and stained for nestin (red). GFP immunostaining (green) labels virus-infected cells. Hoechst (blue) staining labels nuclei. Cells that were co-labeled with GFP and nestin were orange. Images were captured using 40× objective lens. Scale bar: 25 µm. *B,* Quantification of the data showing percentage of nestin^+^ precursors in GFP^+^ population. Three independent experiments were done, each in triplicate and >2000 GFP^+^ cells were counted for each data point.

To examine if phosphorylation on S179 and S208 modulates the pro-neural activity of Neurog1, we infected LeX^+^-enriched rat E13 cortical progenitors with lentiviruses encoding Flag-tagged, wt Neurog1, Flag-Neurog1 SA179/208, dnMEK5, caMEK5 together with wtERK5, or a combination of these constructs as indicated **(** **Fig. 5** **).** Lentiviral GFP was used as a control. All of the viral expression vectors were coupled to GFP through IRES and virus-infected cells were identified by anti-GFP immunostaining (green) **(** **Fig. 5 A** **).** Cortical progenitors were identified by nestin immunostaining (red). Virus-infected cells that express nestin stained orange in merged images. Quantification of the data demonstrated that ectopic expression of Neurog1 decreased the number of cells co-labeled with nestin **(** **Fig. 5 B** **),** suggesting that Neurog1 decreases the pool of cortical progenitors in the infected cell population.

**Figure 6:**
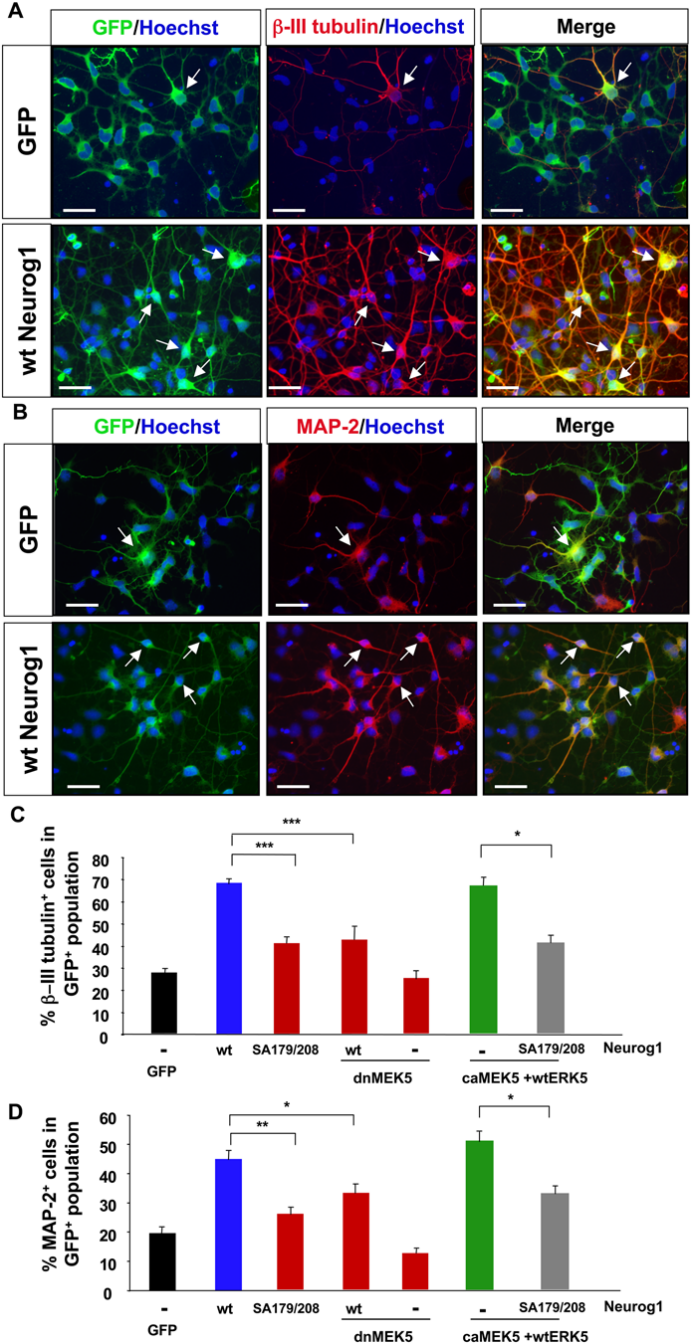
Neurog1-induced neurogenesis is regulated by phosphorylation on S179 and S208 in monolayer cortical progenitor cultures. Cells were cultured as in Figure 5. *A, B,* Representative deconvolution images of cells infected with either GFP control virus or wt Neurog1, and immunostained for β-III tubulin (*A*) or MAP-2 (*B)* (red). Cells that were co-labeled with GFP and β-III tubulin, or MAP-2 were orange. Images were captured using 40× objective lens. Scale bar: 25 µm. *C, D,* Quantification of the data showing percentage of β-III tubulin^+^ neurons *(C),* or MAP-2^+^ mature neurons *(D)*, in GFP^+^ population. Three independent experiments were done, each in triplicate and >2000 GFP^+^ cells were counted for each data point.

Neuronal differentiation was assessed by immunostaining with β-III tubulin and the mature neuron marker, MAP-2 **(** **Fig. 6, A and B** **)**. In contrast to the nestin staining, ectopic expression of Neurog1 increased the number of GFP^+^ cells co-labeled with β-III tubulin **(** **Fig. 6 C** **)** or MAP-2 **(** **Fig. 6 D** **).** The concomitant decrease in nestin expression and increase in β-III tubulin and MAP-2 expression suggest a pro-neural effect of Neurog1. Importantly, the pro-neural effect of Neurog1 was greatly attenuated by co-expression of dnMEK5 **(** **Fig. 5 B** **and** **Fig. 6, C and D** **),** consistent with the data in Figure 1. Significantly, mutations of S179 and S208 to alanines greatly reduced the neurogenic activity of Neurog1. Furthermore, expression of the SA179/208 mutant Neurog1 attenuated the neurogenic activity afforded by ERK5 activation (caMEK5+wtERK5)**.** These data suggest that the pro-neural effect of Neurog1 is regulated by ERK5 phosphorylation and that Neurog1 is a downstream mediator of ERK5's effect on neuronal fate specification.

**Figure 7:**
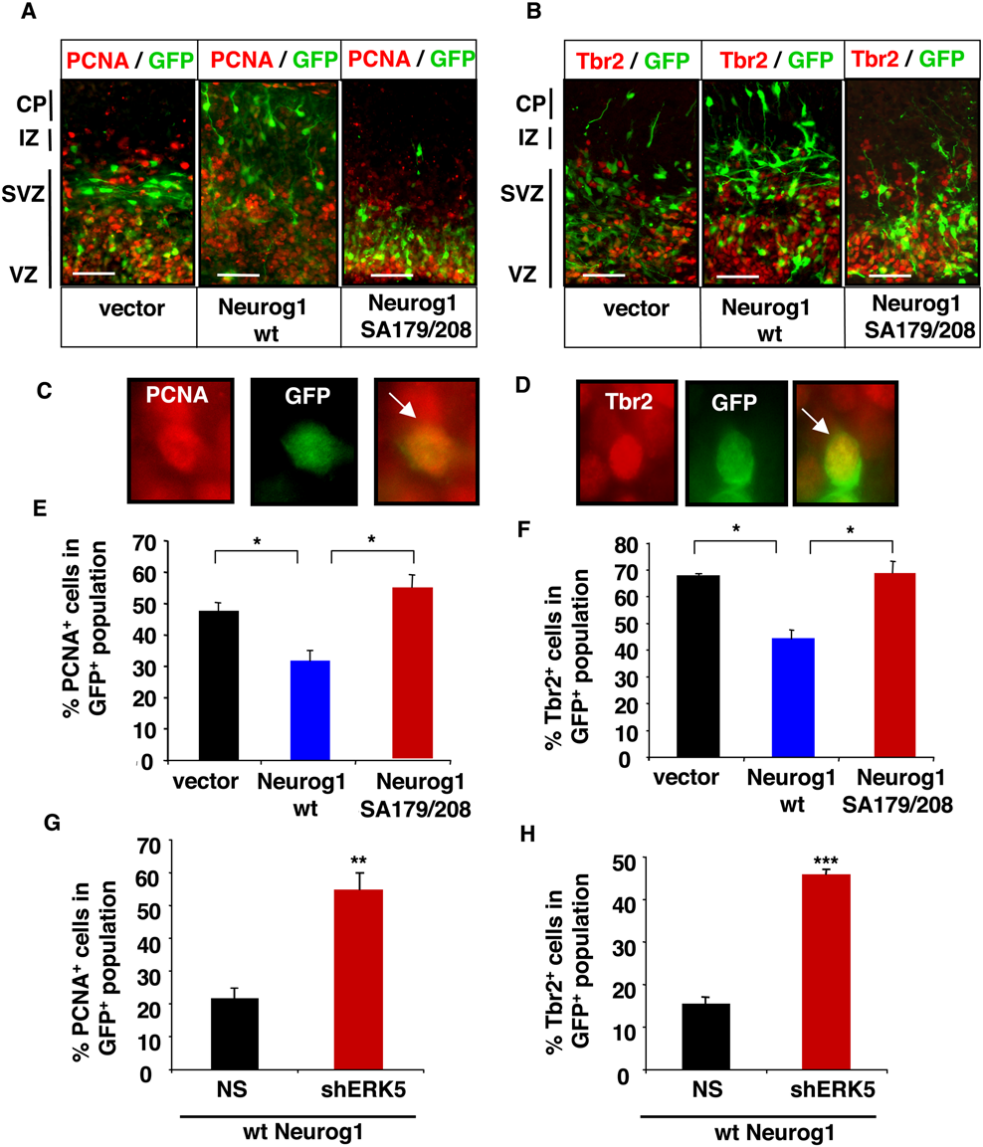
Mutations of Neurog1 at S179 and S208 and inhibition of ERK5 signaling retains Neurog1-transfected cells in proliferating state in organotypic slice cultures. Plasmid DNA encoding control vector, wt Neurog1, SA179/208 Neurog1, shRNA against dsRed (NS) or ERK5 (shERK5) was electroporated *ex vivo* into the dorsolateral telencephalon of rat E15 brain as indicated. A GFP expression vector was co-electroporated to identify transfected cells. Cortical slices were sectioned coronally, cultured for 40 h, and cryosectioned for immunostaining. *A*, *B*, Representative deconvolution images of cortical slices immunostained for GFP (green) and PCNA or Tbr2 (red), respectively. Images were captured using a 20× objective lens. Scale bar: 50 µm. *C, D,* Representative high magnification (63×) images of GFP^+^ cells co-labeled with PCNA or Tbr2, respectively. *E–H* Quantification of cells double-immunostained for GFP and PCNA (panels E and G) or Tbr2 (panels F and H) in total GFP^+^ cells. Vector: vector control. The data were obtained from at least three sections each from three independent experiments.

**Figure 8:**
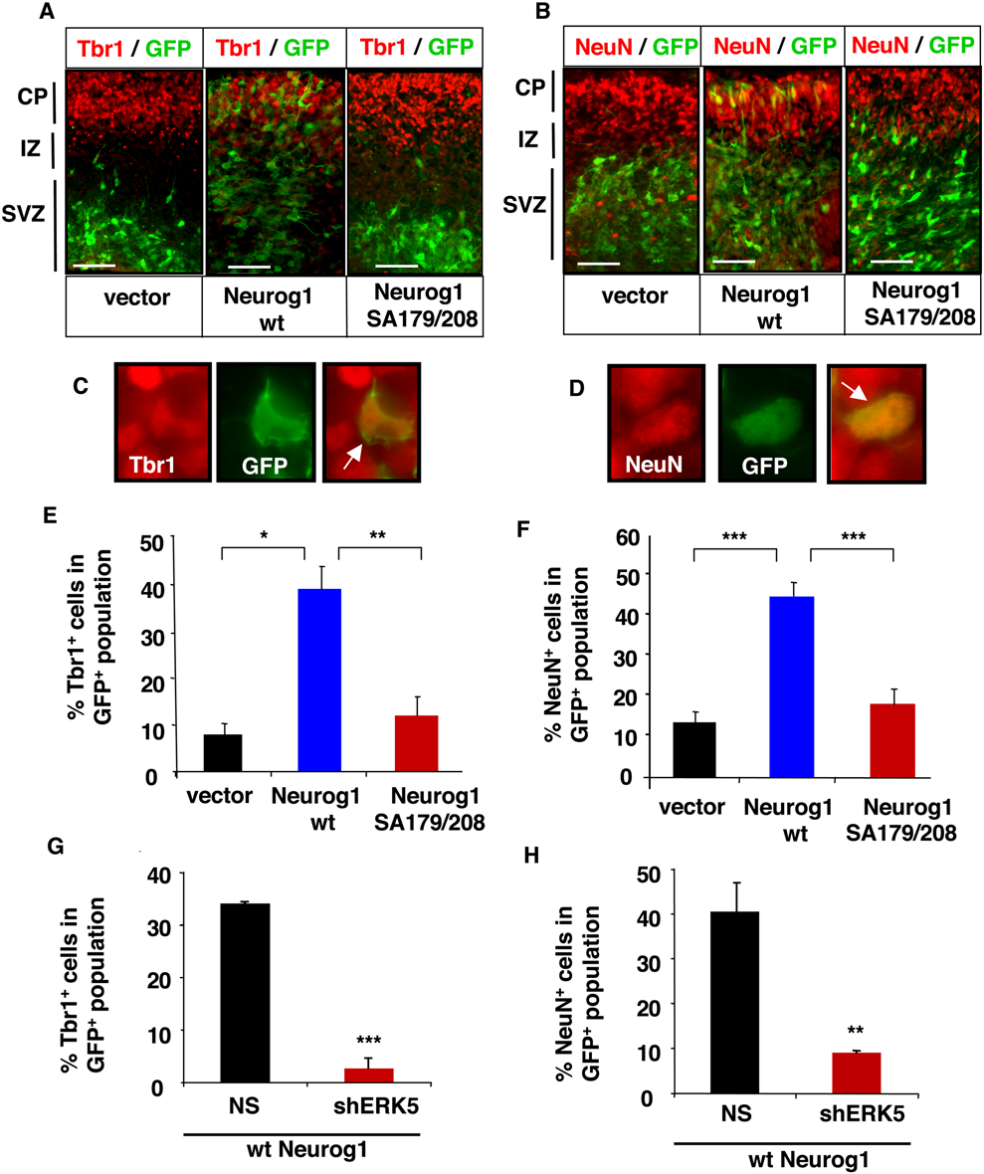
Neurog1-induced neurogenesis is suppressed by mutations at S179 and S208 and inhibition of ERK5 signaling in organotypic slice cultures. Cortical slices were transfected and cultured as in Figure 7. *A*, *B*, Representative deconvolution images of cortical slices immunostained for GFP (green) and Tbr1 or NeuN (red), respectively. Images were captured using a 20× objective lens. Scale bar: 50 µm. *C,D,* Representative high magnification (63×) images of GFP^+^ cells co-labeled with Tbr1 or NeuN, respectively. *E–H* Quantification of cells double-immunostained for GFP and Tbr1 (panels E and G) or NeuN (panels F and H) in total GFP^+^ cells. Vector: vector control. The data were obtained from at least three sections each from three independent experiments.

We utilized *ex vivo* electroporation coupled to organotypic slice culture to examine the effect of SA179/208 mutations on Neurog1's pro-neural activity. The organotypic slice cultures maintain some of the anatomy and cell-cell interactions of the intact cortex [21]. Plasmid DNA encoding vector control, wt Neurog1 and the Neurog1 SA179/208 mutant were injected into the lateral ventricles of dissected E15 rat brains. A GFP plasmid was co-injected as a marker to identify transfected cells. The electrodes were placed in a way to consistently favor plasmid DNA electroporation into the dorsolateral cortex. The cortices were sliced into 300 µm sections and cultured *ex vivo*. The cellular phenotype of the transfected cells (GFP^+^) was identified by immunostaining for PCNA **(** **Fig. 7 A** **)**, a marker for cells in early G1/S phase, or the T-box brain (Tbr) 2 **(** **Fig. 7 B** **),** a transcription factor and marker for cells actively proliferating in the upper layer of the ventricular zone (VZ) and the sub-ventricular zone (SVZ) [22], [23]. The slices were also stained with Tbr1 **(** **Fig. 8 A** **)** or NeuN **(** **Fig. 8 B** **),** markers for post-mitotic neurons in the cortical plate during development [22], [24], [25] and mature neurons, respectively**.** Co-labeling of cells immunopositive for GFP and PCNA (**Fig. 7 C** **)**, Tbr2 (**Fig. 7 D** **)**, Tbr1 (**Fig. 8 C** **)**, or NeuN (**Fig. 8 D** **)** was confirmed using de-convolution imaging under high magnification.

In control, vector-transfected cells, most of the transfected cells (GFP^+^) were still proliferating (47% PCNA^+^ or 66% Tbr2^+^) **(** **Fig. 7, E and F** **)** and localized to SVZ/VZ. Only a small fraction of the cells had differentiated (8% Tbr1^+^ or 14% NeuN^+^) **(** **Fig. 8, E and F** ***)*** and migrated to the cortical plate (CP) after 40 h in culture**.** However, more of the wt Neurog1-transfected cells were found in the CP layer and were Tbr1^+^ (37%) or NeuN^+^ (45%). This supports our *in vitro* cell culture data shown in Fig. 1, 5, and 6 and demonstrates a role for Neurog1 in promoting neurogenesis in slice cultures. In contrast, cells transfected with the Neurog1 SA179/208 mutant behaved like vector control-transfected cells; most remained proliferative (54% PCNA^+^, 68% Tbr2^+^) and only a few expressed the post-mitotic neuron markers Tbr1 (12%) or NeuN (18%). Because the Neurog1 SA179/208-transfected cells found in the SVZ/VZ expressed PCNA and layer specific marker Tbr2 but did not express post-mitotic neuron markers Tbr1 or NeuN, we conclude that Neurog1 phosphorylation at S178 and S208 does not affect neuronal migration. These data suggest that mutations at the putative ERK5 phosphorylation sites S179 and S208 suppress the pro-neural activity of Neurog1.

We also examined the effect of blocking ERK5 expression on the pro-neural activity of Neurog1 using the organotypic slice culture assay. To block expression of endogenous ERK5, we constructed a retroviral shRNA vector against ERK5. A non-specific shRNA against dsRed (NS) was used as a control. Specific knockdown of ERK5 expression by shERK5 was confirmed in cultured rat E13 cortical progenitors **(** **Fig. S1** **)**. Cells co-transfected with Neurog1 and shERK5 had greatly increased numbers of proliferative PCNA^+^ or Tbr2^+^ cells (**Fig. 7, G and H**) and fewer differentiated Tbr1^+^ or NeuN^+^ neurons (**Fig. 8, G and H**) compared to those co-transfected with Neurog1 and NS control. These data suggest that blocking ERK5 expression and signaling attenuates the pro-neural effect of Neurog1 in cortical slice cultures.

## Discussion

The objective of this study was to investigate downstream mechanisms mediating the neurogenic activity of ERK5. We published evidence that ERK5 is highly expressed in proliferating cortical progenitor cells and is both necessary and sufficient to specify cortical progenitor cells towards a neuronal fate [18]. We report here that Neurog1 is a downstream target of ERK5. ERK5 directly phosphorylated Neurog1 *in vitro* and modulated the transcriptional and pro-neural activity of Neurog1 in cortical progenitors. We also identified S179 and S208 as putative ERK5 phosphorylation sites on Neurog1. These two serine residues are located within the putative transactivation domain of Neurog1 [26]. Intact S179 and S208 were required for Neurog1's function since replacing each with a non-phosphorylatable alanine greatly attenuated the ability of Neurog1 to initiate transcription and specify neuronal fate. These data identify Neurog1 as a downstream target mediating the pro-neural effect of ERK5 and implicate phosphorylation of Neurog1 as a novel mechanism regulating neuronal fate commitment of cortical progenitors.

During cortical neurogenesis, the pro-neural bHLH transcription factors including Neurog1, Neurog2, and Ascl1 direct cortical progenitors to a neuronal fate [9]. Many signaling pathways have been implicated in stimulating neuronal differentiation including the Wnt/β-catenin pathway [27], PI3K [28], Notch pathway [15], [29], [30], and the ERK1/2 pathway [31], [32]. However, it is not known if these signaling pathways regulate the pro-neural activity of the bHLH transcription factors. Protein phosphorylation has been implicated in regulating the stability and function of bHLH transcription factors during neuronal terminal differentiation, maturation and sub-neuronal phenotype specification. For example, Neurog1 stability is regulated by protein phosphorylation and subsequent ubiquitin-mediated proteolysis [33]. The function of Xenopus NeuroD in retinal neuron differentiation is inhibited by glycogen synthase kinase (GSK) 3β, presumably via GSK3β phosphorylation of XNeuroD [34]. CaMK II induces the phosphorylation of NeuroD at Ser 336, which regulates granule neuron dendritic morphogenesis during cerebellar development [35]. In addition to their pro-neural activity, Neurog1 and Neurog2 also regulate neuron migration [36], [37], and a recent report implicates phosphorylation of Neurog2 in the regulation of neuron migration [37]. Another recent report demonstrates that Neurog2 phosphorylation at Ser231 and Ser234 by GSK3 regulates the specification of motor neuron subtypes but has no effect on the total number of neurons produced *per se* [38]. There is little published data addressing the role of kinases or phosphorylation in modulating the production of neurons which can be attributed to the function of Neurog1, Neurog2, or Ascl1. Our study is the first to demonstrate that phosphorylation of Neurog1 modulates the total number of neurons produced from cortical progenitors.

The putative ERK5 phosphorylation sites S179 and S208 are evolutionarily conserved among mouse, rat, and human sequences of Neurog1 **(** **Fig. S2** **)**. A putative phosphorylation site similar to S208 is also found in the Neurog1 sequence of non-mammalian vertebrates zebrafish and xenopus. Furthermore, two putative phosphorylation sites comparable to S179 and S208 exist in the Neurog2 sequence **(** **Fig. S3** **).** Therefore, protein phosphorylation of the pro-neural bHLH transcription factors may be a common mechanism by which extrinsic factors in the neurogenic niche regulate the neuronal fate specification of neural progenitor cells.

A large body of evidence suggests that environmental cues such as the microenvironment surrounding progenitor cells play an important role in cell fate determination of neural progenitor cells [16], [17], [39]–[42]. Since ERK5 is activated by neurotransmitters, growth factors, and neurotrophins including NT3/4 and BDNF [43], [44], it seems likely that environmental cues may instruct cortical progenitors to become neurons by activating the ERK5-Neurog1 pathway.

Interestingly, the Neurog1-NeuroD axis bears significant similarity to the myogenic MyoD-Myogenin pathway of muscle differentiation [45]. There is evidence that the myogenic bHLHs, MyoD and Myogenin, are phosphoproteins [46], [47] and that MyoD and Myogenin can be directly phosphorylated by ERK5 [48]. Besides its high level of expression in the nervous system, ERK5 is also highly expressed in muscle and is required for muscle differentiation [48]. Thus, phosphorylation of the bHLH transcription factors may be a conserved mechanism by which ERK5 regulates muscle and neuron differentiation. Neurog1 also confers anti-gliogenic activity independent of its pro-neural activity in the nervous system [7]. It would be interesting to examine if ERK5 phosphorylation also modulates the anti-gliogenic properties of Neurog1.

A number of ERK5 substrates have been identified, including myocyte enhancer factor (MEF) 2C, Sap 1a, c-myc, SGK (serum- and glucocorticoid-inducible kinase), the pro-apoptotic Bcl-2 family protein BAD, and pp90Rsk [20], [49]–[51]. ERK5 has been implicated in many aspects of cellular and physiological function including apoptosis, cell cycle, muscle differentiation, cardiovascular function, neuronal survival, and neuronal cell fate specification [18], [50]–[52]. Data presented here identify Neurog1 as a new substrate for ERK5 and implicate ERK5 in the regulation of the pro-neural bHLH transcription factors.

In summary, we identified a novel mechanism during cortical neurogenesis in which the pro-neural and transcriptional activity of Neurog1 is regulated by ERK5 through phosphorylation. Similar kinase phosphorylation mechanisms may also regulate the pro-neural activities of other bHLH family transcription factors including Neurog2 and Ascl1.

## Materials and Methods

### Reagents

The following plasmids have been described: the lentiviral transfer vector pRRL-cPPT-CMV-X-PRE-SIN [53], a kind gift from Dr. W. Osborne (University of Washington); NeuroD2-Luc reporter [3]; expression vectors for dnMEK5, caMEK5, wtERK5 and dnERK5 [20]. The Neurog1 expression vector (pCS2-NeuroD3) and the 3xE-box-Luc reporter (pCS2-EB7-Luc) were obtained from Dr. Jim Olson [3]. The cDNA sequence of Neurog1 was sub-cloned into pcDNA3 with a Flag-tag added to its N-terminus. For the truncated wt GST-Neurog1 and SA179/208 mutant, the cDNA sequences corresponding to residues 151–244 were subcloned into the pGEX vector. The rabbit polyclonal anti-ERK5 antibody [43] and the polyclonal Tbr2 and Tbr1 antibodies have been described [22]. The rabbit polyclonal anti-Neurog1 antibody was generated by immunizing rabbits (Cocalico Biologicals, Reamstown, PA) with GST-Neurog1 fusion protein. The following antibodies for immunostaining were purchased commercially: mouse monoclonal (M1) anti-Flag antibody (Sigma, St. Louis, MO); mouse monoclonal anti-nestin (Becton Dickinson, Bedford, MA); mouse monoclonal anti-NeuN (Chemicon, Temecula, CA); rabbit polyclonal anti-GFP (Molecular Probes, Eugene, OR); mouse monoclonal anti-GFP (Molecular Probes), mouse monoclonal anti-β-III tubulin (Promega, Madison, WI); mouse monoclonal anti-MAP-2 (Sigma); monoclonal anti-LeX (CD15 FITC) (Becton Dickinson); mouse monoclonal anti-PCNA (Chemicon). The following inhibitors were purchased commercially: Proteasomal Inhibitor Cocktail Set (Calbiochem, San Diego, CA), pan-caspase inhibitor ZVAD-FMK (R&D Systems, Minneapolis, MN).

### Lentivirus constructs

We have constructed lentiviral transfer vectors as previously described [18]. All genes of interest were N-terminal Flag-tagged and inserted into a multiple cloning site upstream from an IRES-directed marker protein eGFP (enhanced green fluorescent protein).

### Retrovirus constructs

The shRNA sequences against mouse ERK5 and dsRed [54] were cloned into the BamHI/Xbal sites of the multiple cloning site of the pSIE dual promoter retroviral vector [55]. The shRNA expression is under the control of human U6 promoter and GFP expression is under the control of the EF1α promter. The targeted sequences used were as follows: ERK5 (aa 106–111) acacttcaaacacgacaat; dsRed-C1 agttccagtacggctccaa.

### Rat E13 cortical progenitor cell cultures

These were prepared as described and cells were maintained in culture medium containing 10 ng/ml bFGF (Invitrogen, Inc) [18]. For the adherent culture monolayer assay or progenitor cell clonal assay, cortical progenitors were enriched by magnetic activated cell sorting (MACS) after labeling with an antibody against LeX (anti-CD15), a cortical progenitor marker [56]. For the neurosphere assay, freshly dissociated E13 cortical progenitor cells were plated at a clonal density of 2000 cells/ml in petri dishes without any coating.

### *Ex vivo* electroporation and organotypic slice culture

Plasmid DNA was injected into the lateral ventricles of E15 rat brain and electroporated into the cortex using a CUY21 Edit square pulse electroporator (Bex Co. Ltd., Japan). Plasmid was targeted to the dorsal region of the telencephalon by placing the positive electrode directly superior to the telencephalon and the negative electrode ventral to the head. Following electroporation, dissected cortices were immobilized in a 4% agarose mold and sliced into 300 µm slices using a vibrating microtome, transferred to permeable membranes and placed in growth medium for 40–50 h. Organotypic slice cultures were fixed and cryosectioned into successive 20 µm slices for immunostaining. For Western analysis, regions with GFP expression (green) were micro-dissected out under a fluorescence microscope and homogenized with a syringe in lysis buffer followed by vortexing to prepare cell lysates.

### Luciferase reporter gene assays

Rat E13 cortical progenitor cells were transiently transfected at days in vitro (DIV) 2 using the Nucleofector® electroporation method per manufacturer's instruction (Amaxa Biosystems, Inc.). Briefly, the cells were grown as a monolayer in coated plates for 1–2 d, trypsinized at room temperature for 5 min and centrifuged at 735*g* for 5 min. Cells were resuspended in Rat Neural Stem Cell Nuclofector® Transfection Reagent (Amaxa Biosystems, Inc.) at a density of 3×10^6^ cells/100 µl. For each transfection, 3×10^6^ cells were transfected with total plasmid DNA not exceeding 10 µg using the A31 (low toxicity) protocol. Following electroporation, cells were resuspended in pre-warmed (37°C) regular culture medium and incubated at 37°C for 20 min. Cells were then resuspended in regular culture medium and plated onto 24-well plates coated with laminin and poly-D-lysine (PDL). Cell lysates were prepared 36–48 h later for reporter gene assay as described [43].

For E16 cortical neuron luciferase reporter gene assays, neurons were prepared and transfected using LipofectAMINE 2000 (Invitrogen) as described [43], [52].

### Calf intestinal alkaline phosphatase treatment

Protein lysates were homogenized in protein lysis buffers lacking phosphatase inhibitors [57]. One hundred micrograms of protein lysates were treated with 10 units of CIP (Fermentas, Inc.) and 10 mM MgCl_2_ for 60 min at 37°C. For control, protein lysates were homogenized in regular protein lysis buffers containing phosphatase inhibitors.

### *In vitro* kinase assays

*In vitro* ERK5 kinase assays were performed as described [43], [58]. Briefly, whole cell lysates (1000 µg protein) were incubated at 4°C for 2.5 h with 6 µl of polyclonal anti-ERK5 antibody. Protein A-Sepharose beads (60 µl) were then added, and the mixture was incubated at 4°C for an additional hour. The activity of ERK5 in the immunoprecipitates was quantified by a kinase assay using 30 µg recombinant wt GST-Neurog1 (151–244) or GST-Neurog1 SA179/208 (151–244) as the substrates. Relative radiolabeled Neurog1 and ERK5 was quantified using autoradiography and normalized to total wt GST-Neurog1 and GST-Neurog1 SA179/208 protein levels.

### Microscopy and image acquisition

Representative images were generated by a Marianas imaging system (Intelligent Imaging Innovations, Inc.) incorporating a microscope (Axiovert 200M; Carl Zeiss MicroImaging, Inc.) with an X,Y motorized stage, shuttered 175 W xenon lamp coupled with a liquid light guide, a digital camera (CoolSNAP HQ; Roper Scientific), and 20× or 63× objective lens (Axiovert; Carl Zeiss MicroImaging, Inc.) as indicated. Slidebook software package was used for system control and image processing. Adobe Photoshop was used to uniformly optimize images.

To capture images for quantification of organotypic slice cultures, images were generated with an inverted fluorescence microscope (Leitz DMIRB; Leica) using a 40× objective lens (Leitz; Leica). MagnaFire digital microscope camera and MagnaFire software (Optronics, Inc.) were used for system control and image processing.

### Cell counting of organotypic slice sections

For quantification**,** 20 µm sections were chosen that expressed comparable levels of GFP within the same region of the dorsolateral telencephalon. For each condition, photographic images were generated from three separate transfected regions using a 20× or 40× objective. To quantify the number of transfected cells that co-labeled with the cell-specific markers, each digital image was segmented into one-inch horizontal bins. The total number of cells per bin were recorded by Hoechst (blue channel) staining, the total transfected cells per bin were recorded by GFP (green channel) immunostaining, the total number of cells labeled for the cell-specific markers (PCNA, Tbr2, Tbr1, or NeuN) per bin were recorded (red channel), and the total transfected cells which co-labeled with the cell-specific markers (red and green channel) were recorded and cross-referenced with the Hoechst. To ensure that cells were within the same plane in the digital images, each co-labeled cell was confirmed by toggling back and forth between the blue, red, green, and red-green channels in Adobe Photoshop. To validate cell counting acquired from 20× or 40× images, co-labeled (green/red) cells were visualized at higher magnification (63×) using a Marianas Imaging system and deconvolution scope.

### Statistical Analysis

All data are expressed as the mean±standard error of means (SEM) from at least three independent experiments (n≥3). Pair-wise comparisons between means were tested by a Student's t-test, two-tailed analysis. n.s. not significant; *p<0.05; **p<0.01; and ***p<0.001.

## Supporting Information

Figure S1 (0.36 MB TIF)

Figure S2 (5.26 MB TIF)

Figure S3 (1.64 MB TIF)
