# Supplementary figures and images for: ERK5 MAP Kinase Regulates Neurogenin1 during Cortical Neurogenesis
Source: PLoS One. 2009 Apr 13;4(4):e5204. doi: 10.1371/journal.pone.0005204 (PMC2664926; doi:10.1371/journal.pone.0005204)

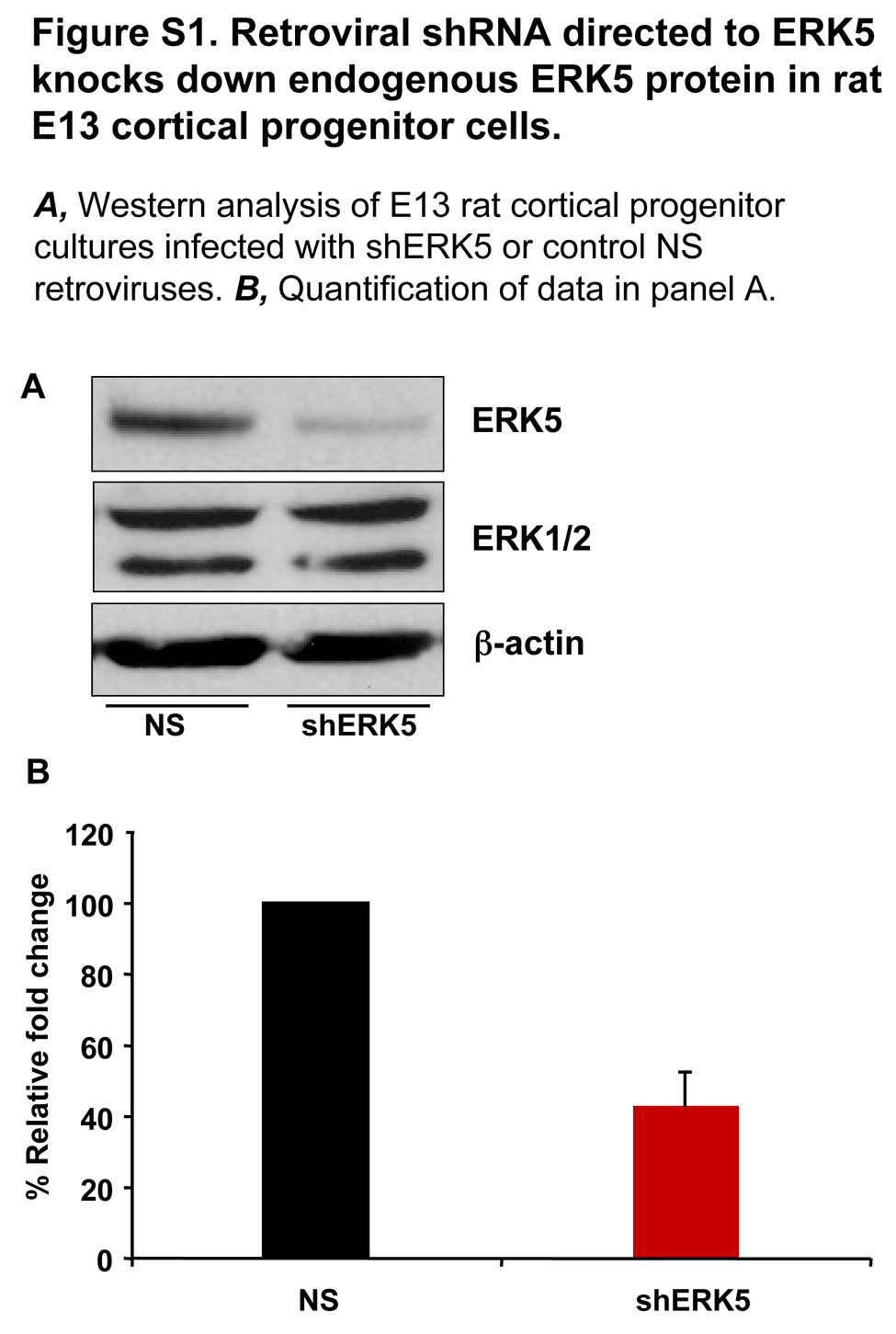

Supplement: Figure S1 — (0.36 MB TIF) [file pone.0005204.s001.tif]

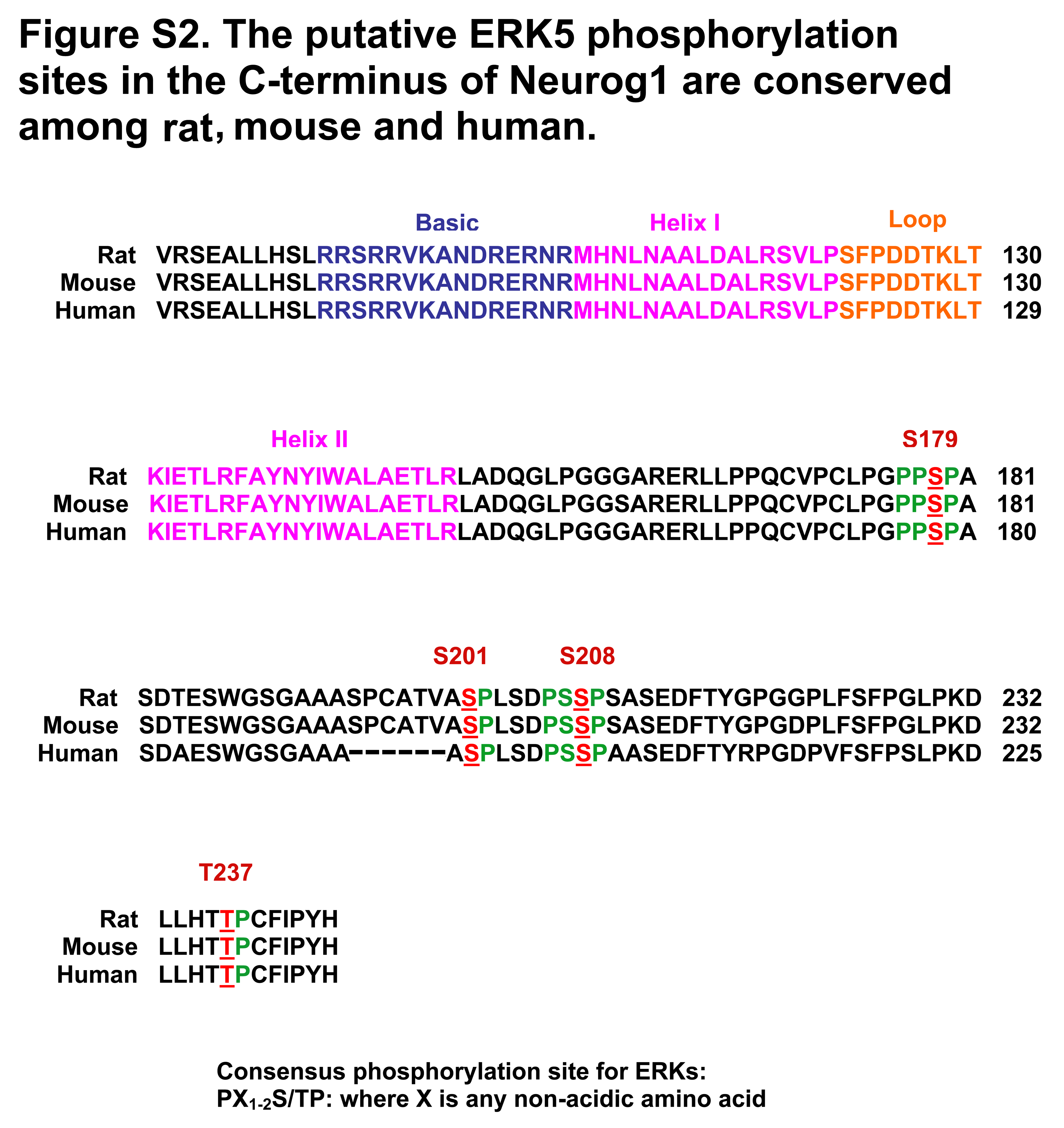

Supplement: Figure S2 — (5.26 MB TIF) [file pone.0005204.s002.tif]

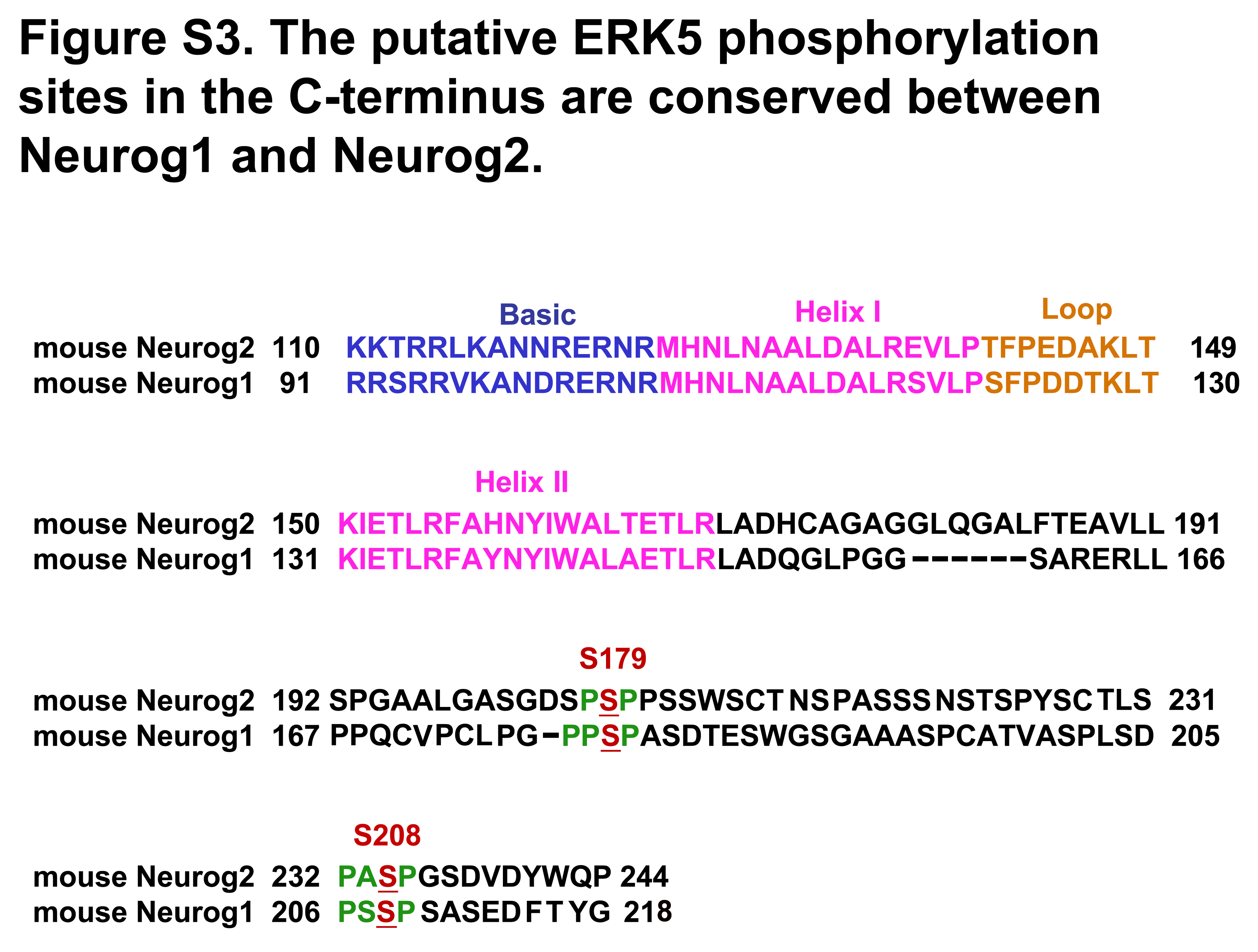

Supplement: Figure S3 — (1.64 MB TIF) [file pone.0005204.s003.tif]
